# Intercultural therapy with Ultra-Orthodox Jews in Israel: the complexity of the encounter between secular therapists and Haredi clients

**DOI:** 10.3389/fpsyg.2024.1356242

**Published:** 2024-06-19

**Authors:** Einat Doron, Sławomir Tobis, Jan Domaradzki

**Affiliations:** ^1^Independent Researcher, Binyamina, Israel; ^2^Department of Occupational Therapy, Poznan University of Medical Sciences, Poznań, Poland; ^3^Department of Social Sciences and the Humanities, Poznan University of Medical Sciences, Poznań, Poland

**Keywords:** culture, Haredim, intercultural therapy, secular therapists, Ultra-Orthodox Jews, Israel

## Abstract

This paper explores the exceptional intercultural encounter between secular therapists and Ultra-Orthodox Jews in Israel, focusing on two key aspects. Firstly, it explores the distinctive attributes and conflicts inherent in treating Ultra-Orthodox individuals. On the one hand is the secular Israeli therapist, whose base is in Western philosophy that prioritizes individuality, cultural diversity, and tolerance of differences. On the other hand is the Haredi client, entrenched in values from Jewish tradition and religious principles that amplify solidarity and collectivism while rejecting prevalent secular culture. The existing socio-political climate in Israel often positions these two as potentially conflicting cultures. Secondly, the paper seeks to illuminate the uncommon dynamics of the minority-majority power balance within the therapeutic relationship. In contrast to prevalent literature in intercultural therapy, which typically frames the client as a representative of a disadvantaged minority and the therapist as a representative of a dominant majority, this article aims to unravel a nuanced power balance, where those in the minority perceive the dominant culture both as a threat to its way of life and as a despised entity, but paradoxically rely entirely on its financial support. This reveals a complex and intricate interplay of dominance and dependence, shaping a therapeutic relationship that defies conventional expectations.

## Introduction

Intercultural therapy (ICT) has become a common practice and is expressed in almost every therapeutic session, but it is more evident in societies with strict internal codes such as among the Ultra-Orthodox Jews (Haredim) community in Israel. ICT is a complex and dynamic meeting of personal values concerning world views originating in culture, beliefs, values and emotional biases regarding different ethnic groups, as well as the identity and affiliation group of the therapists themselves (Sue et al., 2009). The ultimate aim of developing such cultural competence should be that, while members of minority groups, i.e., groups with observable characteristics or practices such as religious, ethnic/racial or sexual orientation, feel comfortable seeking help, the service providers are attuned to the nuances of individuals and their culture (Kirmayer, 2011).

This intercultural encounter holds unique attributes when it takes place in Israel’s complex and explosive social-political arena, since the Ultra-Orthodox (UO), although a minority, control many aspects of the general public’s lives and hold positions of great power in the government (Cohen, 2006; Cincotta and Kaufmann, 2009). The UO have strict standards of religious observance, rigid and conservative dress codes and tend to live in separate communities with their own education system, in order to focus on religious precepts and protect themselves from secular influences (i.e., Coleman-Brueckheimer et al., 2009).

Although change is evident toward the secular Western system of cultural values and norms which prioritize individuality, cultural diversity, and tolerance of differences on which psychotherapy is grounded (Finkelman, 2014; Gopalkrishnan, 2018; Koç and Kafa, 2019), and the concepts and practice of psychotherapy itself (Caplan and Stadler, 2012; Freund and Band-Winterstein, 2013, 2017; Schnall et al., 2014; Doron, 2020; Keidar et al., 2021, 2022), a good deal of suspicion, lack of trust and even hostility toward the secular general public remains, which, in turn, often holds great contempt of the UO community, due to inequality in contributions to the country’s economy and military service (Greenberg and Witztum, 2013; Finkelman, 2014).

At the same time, while previous studies have focused either on the premises and goals of ICT with UO clients or described psychological encounters from the Haredim’s perspective (Bilu and Witztum, 1993; Bilu and Witztum, 1995; Margolese, 1998; Keidar et al., 2022), much less attention tends to be paid to the secular therapists’ experiences. Even studies that do describe the ICT from the therapists’ perspective also often omit their personal experiences, needs and the distress that results from working with a UO client, and focus on their opinions regarding the Haredim’s needs (Band-Winterstein and Freund, 2015; Freund and Band-Winterstein, 2017; Haimovich and Leiser, 2017; Keidar et al., 2021, 2022). This paper therefore aims to highlight the complexity of the encounter between secular therapists and Haredi clients. In contrast to prevalent literature in intercultural therapy, which typically frames the UO client as a representative of a disadvantaged minority and the secular therapist as a representative of a dominant majority, this article also seeks to unravel a more nuanced power balance.

### The Ultra-Orthodox Jews in Israel

The UO Jews, also known as Haredim (sing. Haredi), constitute 13.3% of Israel’s Jews (Malach and Cahaner, 2022). They are characterized by their strict adherence to Jewish religious law, which covers every aspect of daily life. The Haredim maintain a cultural distance from (and often hostility toward) the surrounding wider secular society. In order to avoid desecrating the holy Hebrew language they also distinguish themselves by speaking Yiddish, a language used by Jews in central and Eastern Europe before the Holocaust. Television, cinema, and the use of the internet for anything other than work is forbidden. The use of Smartphones with a censored kosher internet is allowed in some but not all UO communities (Hoffman and Ben Shalom, 2014). The different UO groups of the community differ, among other things, by their disposition to disclosure and to receiving professional treatment from the wider secular society (Goodman and Witztum, 2002).

The average UO family has six children and many have up to 10 (Zicherman, 2014). The education system is also completely different from that of the Western world. UO children are brought up to develop a reverence for their parents, with whom their relationship will tend not to be amicable. Parents avoid showing their feelings toward their children, especially in public, and they display self-control and practicality. They avoid calling each other by their first names in order to teach their children respect. Since this community is undergoing many changes, however, the nature of the children’s upbringing is gradually becoming more open and less restrained (Zicherman, 2014).

The UO community is also characterized by strict gender role division: men are expected to take part in the society of scholars of religious studies in Yeshiva and the burden of income falls on women. Many women go out to work in the knowledge that the community encourages a livelihood rather than a career, and that they are expected to do the housework (Caplan, 2017).

Given this community’s nature, from a small self-segregated group, the UO population is expected to constitute a fifth of the population (Malach and Cahaner, 2018). This demographic factor is complemented by the economic component; the poverty rate amongst the UO is over 50 % and the average family income is 50 % lower than the Israeli average. This became significant with the acceptance of Israel as a member of the OECD (Zicherman and Cahaner, 2012; Malach and Cahaner, 2018).

At the same time, over the past two decades there has been a notable shift in the integration of the Haredim into Israeli society, despite their initial resistance to secular lifestyles. This transformation is evident in the increasing participation of UO men and women in higher education and the workforce (Malach and Cahaner, 2022).

Similarly, although it has been established that encounters between Haredi Jews and non-Haredi therapists may benefit both parties (Freund and Band-Winterstein, 2013, 2017; Doron, 2020; Keidar et al., 2022), potential misunderstandings or even conflicts continue to proliferate (Finkelman, 2014). These result from a general tension between the Haredi religion and the traditionalist-collectivist culture, and non-Haredi therapists’ secular worldview. While the former may find it difficult to discuss some issues with secular therapists, who are often perceived as *strangers* who cannot be trusted (Freund and Band-Winterstein, 2013, 2017), the latter may consequently find it difficult to adapt to the Haredim’s way of communication and may feel that their professional authority is being questioned.

### The Ultra-Orthodox and the secular

Despite a shared commitment to Israel as a Jewish homeland and refuge, divisions between the Haredi minority and the secular majority persist. These divisions are reflected in starkly contrasting positions on many questions of public policy, including marriage, divorce, military conscription, gender segregation and public transport. The UO express the view that Israel’s government should promote religious beliefs and values, while secular Jews strongly favor the separation of religion from government policy. A 2016 survey revealed that secular Jews in Israel are more uneasy about their children marrying a UO than a Christian (Pew Research Center, 2016).

Beyond the differences in their attitudes regarding keeping Jewish laws, secularism is perceived by the UO as a cultural opposite: ultra-orthodoxy lifts the spirit and secularism the body; ultra-orthodoxy suppresses the lusts and secularism develops them; ultra-orthodoxy honors the elderly and secularism worships the young. These are indeed two opposite worlds. The Haredim clearly feel that the two may not be integrated (Zicherman, 2014).

The Haredim suffer no inferiority complex in relation to the excesses of secular materialism regardless of what secular thinking might commonly have us believe. For many UO a life of poverty and asceticism is an ideological choice, placing spirituality as an opposing ethos to the materialism they despise. Not only is Western materialism unappealing to them, but they are disdainful of it. The Haredim are scornful of secular Jews (Zicherman, 2014). They see themselves as the last sane rational group still active in an absurd, twisted world (Brown, 2017). The Haredi culture thrives on a clear dichotomy, rejecting the *other* from an early age, with no middle ground. This binary perspective supported by rabbinical ideology portrays the *other* with contrasting values, behaviors and discourse, reinforcing the Haredi way of life as morally superior (Sagi, 2006; Elimelech, 2012 in Zicherman, 2014).

The disputes and disdain of secular Jews and UO Jews began many years ago, but, since the UO sector in Israel has become substantially larger in number, the issue of the diverted groups has moved to the center of public discourse. One of the reasons for this is that the majority of married UO men neither work nor serve in the army (Greenberg and Witztum, 2013; Finkelman, 2014). For most of their married life they attend studies in religious schools which are subsidized by the government. The UO contribute little in terms of tax revenue and tend to remain below the poverty line, availing themselves of welfare assistance. Since UO families are large, they also receive government-sponsored child benefit (Cincotta and Kaufmann, 2009; Finkelman, 2014; Bagno-Moldavsky, 2016). These benefits are mostly financed by the secular sector, which recently has begun to resist (Zicherman and Cahaner, 2012; Zicherman, 2014). While secular Jews financially support the UO community, they are also often perceived by a large proportion of the Haredim as promiscuous, poorly educated and in thrall of materialistic values, or even as a threat to traditional UO culture and lifestyle (Caplan, 2006, 2007).

As well as the economic aspect, there is also politics. The unique situation in Israel means that, unlike other subcultures and minorities, rather than being rebuffed by influential sources the Haredim have been granted the status of powerful key players in the political arena (Cohen, 2006) and are well-integrated in influential positions of power (Cincotta and Kaufmann, 2009). UO rabbis control, for instance, marriage, conversion and burial certificates of approval, effectively determining the status of seculars’ private lives (Cincotta and Kaufmann, 2009).

At the same time, while these reasons have caused secular Jews to develop a skeptical, sometimes negative attitude toward UO, most of the secular sector knows surprisingly little about Haredim. The social perception of the Haredim is also often constructed by the media, which focus on the above differences and conflicts (Zicherman, 2014). There therefore seem to be no similarities, no common ground for dialog between the two sectors and no will to establish any (Zicherman, 2014).

The UO community, however, is undergoing changes, frequently crossing boundaries as even the most extreme members must avail themselves of secular services (Caplan, 2017; Haimovich and Leiser, 2017). Haredi Judaism is evolving to embrace diversity, engage with modern technologies and media, adopt consumer and leisure culture, and integrate professional discourses in therapy, medicine and psychology (Finkelman, 2014).

### Ultra-Orthodox Jews and psychotherapy

The UO Jews tend to perceive psychology as a method to undermine the very fundamentals of religion, or even seek to replace them with its own (Hoffman and Rossman, 2012). Indeed, Freud (1927, p. 43) argued against religion and in *The Future of an Illusion* called it a “neurosis of humanity.” He claimed that both psychoanalysis and religion referred to the human psyche and its troubles, but whereas religion tries to heal through illusion, psychoanalysis seeks to help a person acknowledge reality as it is (Eshet, 2010).

Western liberalism prioritizes individual liberty, independence and autonomy in contrast to the UO worldview, which emphasizes obedience to divine law and rabbinical authority (Frosh, 2004). UO Jews value tradition, conformity and collective identity over personal growth, differing from psychotherapy’s focus on individual enhancement (Haimovich and Leiser, 2017). Voicing personal distress is unfamiliar and considered illegitimate from the perspective of UO Jews (Freund and Band-Winterstein, 2017), which asserts control of the human psyche through religion (Caplan and Stadler, 2012). The freedom offered by therapy clashes with the restrictions imposed by religious law on thought and speech. There is a great deal of anxiety regarding speaking about, or even thinking about, *forbidden topics*. One example of this type of conflict is the question of whether to go to therapy at all. Therapy may be seen as contradicting the belief in God, who is omnipotent, or demeaning religious belief as insufficiently strong (Hess and Pitariu, 2011).

An important commandment instructs against the use of *lashon hara* (‘defamation’). It is therefore forbidden to speak disparagingly about someone, even if what is said is true, as instructed in the verse: “Whoever of you desires life… guard your tongue from evil” (*Psalms* 34: 12–13). Even recounting such defamation without using names is discouraged, for the listeners might be able to determine those involved (*Chafetz Chaim* 3: 4). In a therapeutic context, this commandment may paralyze clients into silence, as described by Doron (2020), who showed that during therapy with a 7-year-old Haredi boy it was problematic to encourage the child to talk about issues that would later be repressed. Keidar et al. (2021) describe this as follows: “The child acts out the thing rather than speaking the thing.” Some UO Jews also wish to be treated with medication alone in order to ensure they may avoid discussing conflicts or risk expressing anger that may give the appearance of dishonoring their parents or their heritage (Rube and Kibel, 2004).

The leading rabbinic authority prohibits psychiatric patients from consulting *heretic* or atheist psychologists or psychiatrists, fearing a clash with religious-cultural norms (Rube and Kibel, 2004). The relationship hierarchy with traditional Jewish law (Halacha) and the rabbi is elevated above therapy (Freund and Band-Winterstein, 2013, 2017), and introduces continuous tensions and conflicts within the therapeutic dynamic, such as conflicts of loyalties, the forbidden and the permitted, or between right and wrong (Hess, 2018).

When people in the UO community face difficulties in their lives, in their moods, in their behavior, they will choose to uphold the commandments even more stringently to raise their level of spirituality in order to improve their personal-emotional state. When such actions fail to solve their problems, however, they will turn to therapy. This is often felt as a failure on both a practical and a spiritual level (Hess, 2018). Despite the beneficial results, the improvement and the advantages, the closeness and the dependence in the therapeutic relationship are seen as threatening and broach questions of conscience and values, such as whether it is permitted to have such a close relationship, or, specifically, whether one is permitted to have such a relationship with someone whose knowledge is based on material other than Jewish sources of learning. The dependency also leads to a fear of surrendering, experienced as a crossing of boundaries, a loss of the familiar framework, and the fear of loss of control (Hess, 2018).

On the other hand, in spite of all these challenges therapeutic encounters between Haredi clients and non-Haredi therapists have many beneficial aspects for both parties. A recent study by Keidar et al. (2022), for example, showed that even though UO parents experienced some conflict with art therapists working with their children, they saw them in a positive light and acknowledged their empathy, containment, professionalism and avoidance of judgment. Of equally importance are the benefits therapeutic encounters with the Haredi client may also have for secular therapists. For example Keidar et al. (2021) reported that many therapists acknowledged that working with the Haredim resulted in their personal growth and introspection, and helped them to develop such capacities as humility and patience. It was also suggested that since, as secular Jews they did not belong to Haredi culture, it strengthened their clients’ sense of security and facilitated honesty. Freund and Band-Winterstein (2013) and Keidar et al. (2021) similarly reported that many secular therapists acknowledged that therapeutic encounters with secular therapists resulted in a positive shift in the Haredim’s perception of and attitudes toward therapy.

### A change in the community

As we have seen, the characteristics of this ideological self-segregated group are gradually changing, as the UO community becomes increasingly established, self-confident and well-integrated into influential positions of power in Israeli society. These significant changes have contributed to their increased willingness to embrace both the ideas and practices of the general Israeli culture, which until recently were perceived as “meaningless, aimless, rootless, characterized by vandalism, crime, and psychiatric disorders” (Caplan, 2006). One of the fields of Western knowledge that is being gradually assimilated is that of psychological theory and practice (Caplan and Stadler, 2012). The Haredi population now shows greater awareness and willingness to engage with mental health professionals thanks to encouragement from rabbis and UO leaders, showing greater trust in the therapeutic process (Freund and Band-Winterstein, 2013; Freund and Band-Winterstein, 2017; Keidar et al., 2021, 2022).

In seeking mental help, however, the community strongly prefers orthodox doctors and psychologists, who share their cultural understanding. The increasing availability of UO therapists, while beneficial, raises concerns, as clients often prioritize therapists’ religious affiliations over professional competence. Clients rigorously check therapists’ community ties, rabbi affiliation and level of spirituality, sometimes even before assessing professional competence (Hess, 2018). Here too, some change is occurring and parts of this community feel that sending their child to therapy with a secular therapist is “a price they are willing to pay” so that their child will not struggle as much (Keidar et al., 2022).

## Intercultural therapy

Intercultural therapy (ICT) is a type of psychotherapy in which therapists acknowledge cultural differences between themselves and their clients. This enables therapists to establish a culturally sensitive relationship with clients, based on the usual psychodynamic principles, with the aim of bringing about a change in thinking or behavioral patterns. It is important, however, to note that therapists’ preconceived notions about their clients’ culture may pose a challenge to the therapeutic process (Montgomery et al., 2020). The ultimate goal is for diverse individuals to feel comfortable in seeking help and for service providers to understand the nuances of different cultures (Kirmayer, 2011). Intercultural psychotherapy addresses all aspects of clients’ daily life and social interactions and incorporates a critical perspective toward racial, cultural and social power dynamics as they pertain to the psychotherapeutic relationship at the “interface between the social and the individual” (Cockersell, 2019, p. 99) that honors the complexity of racial and cultural experience (Chang, 2021).

Unlike conventional models ICT provides a space for exploring patients’ social and political life beyond therapy, especially in oppressive contexts, and their subjective responses (Littlewood and Ababio, 2019, p. 3). It is commonly applied in psychotherapy between therapists and clients from different cultures, overtly recognizing and exploring cultural differences as integral to the therapeutic process. ICT encompasses dynamic psychotherapy that considers clients’ entire being, including experiences in their communal life (Kareem, 1992).

### Therapeutic issues and challenges of intercultural therapy

Interventions in ICT, especially cultural competence should influence the course of treatment. Different interventions lead to different outcomes. Aside from considering the processes of change in traditional societies that include a transition from collectivist thinking that values family and community to a so-called Western thinking that celebrates individuality, and the conflicts such a transition may give rise to between for instance, the benefit of the individual and the public, we must also consider cultural values since, these values are often the focus from which to initiate the intervention (Jeraisy, 2013). Culturally competent therapists will consider that a therapeutic intervention that lacks sensitivity to the values and characteristics of clients’ culture and background may lead to severe conflicts between clients and their families, in such a way that may exacerbate the distress and even cause danger (Dwairy, 1998). A culturally sensitive intervention, just as the Western intervention, seeks to promote behaviors that might improve clients’ quality of life (Zoabi, 2015). This does not, however, mean promoting clients’ self-satisfaction or enhancing self-awareness providing a wider range of choices. Instead, intervention strategies adapted to the traditional societies such as the UO encourage clients to nurture the harmony with their surroundings, helping them to avoid conflicts with their families and communities, reducing the probability of possible sanctions. These strategies encourage them to relinquish and avoiding confrontations; they emphasize the social and emotional impacts of clients’ decisions on their families and relatives (Zoabi, 2015).

Hearing clients’ narratives in conjunction with a critical awareness of the way socio-political and economic factors may have an impact on clients’ lives, may help to provide a more holistic understanding of clients’ experience and psyche, greatly enhancing the development of a therapeutic alliance. Ignoring the socio-political and economic realities of clients’ lives leaves us vulnerable to reinforcing inequalities and oppression in society by locating the consequences of structural inequalities within the individual rather than in the power structures that uphold dominant cultural hegemony (Agoro, 2019).

It may be argued that allowing clients to determine the direction of their own counseling and psychotherapy is the central foundation of any therapeutic alliance. This position requires a high level of personal awareness and may be especially challenging when working with clients who hold different beliefs and values from therapists’. Significantly, despite best intentions an absence of this critical awareness will inevitably lead to a form of cultural imperialism (Agoro, 2019). With UO clients this is particularly conspicuous since personal awareness is not always valued and clients view therapists as authorities who need to guide them in what to do. This is what they are familiar with: turning to their Rabbi. Hess (2018) reported her clients saying “I’m waiting for you to direct me. Tell me whether it’s right for me to get divorced.”

There are, of course, also benefits. Opportunities contained in this complex relationship between the two sectors when therapists indicated an increased inclination to share difficulties and seek therapy and advice from not-necessarily Haredi mental health professionals have been mentioned, as well as increased trust in the therapeutic process (Freund and Band-Winterstein, 2017). They also mentioned beneficial aspects of such intercultural encounters as allowing clients to conceal their most inner thoughts, specifically from someone who is not part of the community and, therefore, less likely to judge them, making the therapists feel significant (Doron, 2020; Keidar et al., 2021).

### The therapist’s role in intercultural therapy

The ICT involves dealing with the constant tension between sameness and difference (Flaskas, 2012; Freund and Band-Winterstein, 2013, 2017; Doron, 2020; Keidar et al., 2022). It often necessitates leaving the therapist’s comfort zone and venturing into unexplored territories (Rober and De Haene, 2014). Therapists who work interculturally struggle with theory, phenomenology and competing ideologies, as they sort out universals versus social or culture-specific agendas. The task includes cultural underpinnings of alliance building, listening, power disparities, privilege, recovery and attunement to the discourse on social suffering, belief systems, cultural explanatory models, institutions and sociopolitical frameworks. As complex historical legacies and processes are intertwined with political and social realities, that are entering the therapeutic space (Guzder and Rousseau, 2013).

These skills and attunement are part of a dominant discourse in Western society emphasizing respect for others, cultural diversity and tolerance of differences (Gopalkrishnan, 2018; Koç and Kafa, 2019). In reality, upholding this commitment may be challenging, especially when it clashes with other cherished values, such as social equality, lesbian, gay and bisexual rights, the rights of children or animal rights. Whenever cultural openness clashes with values that fail to respect it, uncertainty and confusion arise. Such fears of cultural imperialism may result in an attitude of cultural relativism that makes it difficult to represent any practice or belief as oppressive or at odds with Western values of equality, personal integrity and freedom (Rober and De Haene, 2014).

This may create a risky situation where therapists will make an effort to avoid being ethnocentric by focusing intensely on the culture of the other while bracketing their own culture. The possibility of tensions or difficulties evoked by the cultural differences immediately being considered by therapists as their problems, possibly a problem of knowledge also prevail (Rober and De Haene, 2014; Keidar et al., 2021). They refer to these difficulties initially as something they must deal with, as their responsibility, rather than as a shared difficulty or as a responsibility for them and clients (Rober and De Haene, 2014).

In the context of ICT with the Haredi community, research has demonstrated that, since some issues are considered taboo in their religion and the traditionalist-collectivist culture of the UO communities that should not be discussed with secular Jews, Haredi clients may find it difficult to present their problems to a non-Haredi therapist (Freund and Band-Winterstein, 2013, 2017). Some Haredim consequently prefer therapists with UO or religious backgrounds (Keidar et al., 2022). Freund and Band-Winterstein (2013), for example, reported that in order to gain their Haredi clients’ trust and acceptance, secular social workers had to speak in the accepted Haredi style, which might be unfamiliar to them. It was also suggested that one of the biggest challenges faced by non-Haredi social workers working with the Haredim resulted from OU Jews’ collective culture which places group interests over individual and personal concerns. Haredi clients therefore often found it difficult to talk about oneself and were unable to verbalize their concerns and difficulties.

It has also been suggested that secular professionals are often perceived as *outsiders* or *strangers* who are not *one of us* and that such a perception may impede mutual trust, respect and understanding. The rabbi in particular is also expected to participate in patients’ treatment, which may further undermine the therapists’ authority and competence and cause them distress. In some cases, UO clients may also even be discouraged from seeking help from a therapist who is not orthodox (Freund and Band-Winterstein, 2017). They therefore often face conflict between their professional ethos and authority and that of a rabbi, and may experience frustration and helplessness, and feel unable to do their jobs properly (Freund and Band-Winterstein, 2013).

## Discussion

The ICT positions the therapists as those burdened with examining their assumptions, misconceptions and blind spots. This necessitates the exploration of topics such as privilege, power, observation, code-switching, attunement and alliance-building, through multiple areas, such as non-verbal transmissions, linguistic challenges, decision-making paradigms, counter-transference, explanatory models and stereotyping (Guzder and Rousseau, 2013). All these form a part of developing a cultural competency, a skill anticipated by the intercultural therapist, written about and widely discussed over the years. Authors such as Sue et al. (2009), Cockersell (2019), Montgomery et al. (2020), and local writers, such as Caplan (2006, 2007), Greenberg and Witztum (2013), and Zoabi (2015) wrote profusely on the skills of the competent cross-cultural/multi-cultural/inter-cultural therapist and developed awareness to the field through terms such as *cultural sensitivity*, *multicultural competence* and *cultural responsiveness* up to the current phrase symbolizing the zeitgeist: *cultural humility*.

This awareness in the field of cultural competence has gained momentum and importance, and in 2010 the American Psychological Association (APA) issued an amendment to the therapists’ ethics principles, advising them to treat only those clients for whom they have appropriate training, knowledge, experience and supervision (American Psychological Association, 2010).

Throughout the therapeutic discussion on the medical, emotional and psychological treatment of Haredim in Israel over the years there has been a vast body of knowledge regarding the special attributes of treating this demographic. The professional community saw great importance in making these services available to them, taking great steps in learning and adapting their services accordingly (i.e., Eshet, 2010; Hess and Pitariu, 2011; Caplan and Stadler, 2012; Freund and Band-Winterstein, 2013; Greenberg and Witztum, 2013; Freund and Band-Winterstein, 2017).

All this is to try to ensure that the client receives therapy that will result in positive outcomes and the achievement of treatment goals. Together with the significant body of literature on ICT, research deals with the dominance of the West and talks about therapeutic spaces that ultimately raise unintentional colonial power and imperialism (i.e., Guzder and Rousseau, 2013; Agoro, 2019; Cockersell, 2019; Chang, 2021) and is based on a balance of power and hegemony of the Western culture, perceived as “supreme,” when this “dominant” culture fiercely defends its essence (Halstead, 1995).

In this case, although a minority, the Haredim are disdainful of the dominant culture. This unique position rise to resentment in the secular sector (Zicherman, 2014).

Given the secular therapists’ position in ICT, the effort and attunement it necessitates, and this unique balance of power in the social-political situation in Israel, secular therapists are faced with a unique challenge. When there is mutual disdain but the likelihood of working together in public centers and clinics, the intercultural therapeutic encounter takes on extremely different aspects and nuances. A study illustrating such an aspect of the encounter is that of Keidar et al. (2022), stating that some UO parents wanted to be present at their child’s sessions with a secular therapist in order to monitor and *keep an eye* on the therapist so he or she will say nothing inappropriate.

Whether therapists will be able to bear those feelings in a way that enables non-judgmental empathic listening to the client, whether the topic is considered *political*, irrelevant and a disruption to the course of the therapeutic work, or whether the encounter will be such that allows an authentic response toward what the dyad interaction gives rise to? (Nashef, 2008).

This experience may evoke a weak vs. strong relationship that would influence both sides to act as the other might expect in terms of behavior, perceptions, emotions and thoughts, trying to avoid any areas of anxiety for both parties, thus preventing any opportunity for a creative fruitful connection (Frankel, 2002).

The therapeutic relationships in ICT are thus greatly influenced by therapists’ ability to openly discuss in sessions the cultural differences between them and the client. It is insufficient that they just develop an awareness of their varied emotions and achieve cultural competence; they must also have the ability to confront clients with it (Tang and Gardner, 2014). Scholars have found that culturally congruent and culturally responsive verbalizations in therapy had a more positive impact on client outcomes compared to verbalizations that focus on the universality of human experiences (Farook, 2018).

The concept of genuine relationships in the therapy room emphasizes the importance of each participant, client and therapist, *to be who they are* in therapy, with the other. The real relationship thus emphasizes the value in treatment for participants to see each other authentically person-to-person without the distortions that come from stereotypes and biases. Gelso (2011) contrasts genuineness with *phoniness*, which may happen when either the therapist or the client feels the need to put on an appearance, for example, out of fear of being judged or humiliated. Therapists might experience difficulty in *being who they are* with clients due to over-examining the cultural context and the above-discussed parameters, and thus the relationship might be inauthentic (Gelso, 2011; Keidar et al., 2021). This difficulty was illustrated in a case study by Doron (2020) in which a Haredi boy had scientific questions about the world and nature and the therapist faced the dilemma whether she should give him answers to his questions, satisfying what she thought was a naturally curious nature, thus jeopardizing her position, or whether she should comply with the codes of the UO community and give him the answer “That’s God’s will”?

In a study conducted by Keidar et al. (2021) these concerns of the secular art therapists’ stance in the dyad were clearly shown. Therapists’ criticism of the Haredi society emerged while treating UO children. When displays of racism and hierarchical thinking appeared in sessions, they felt deeply uneasy, feeling that these were not the notions of a just society. When they felt the contempt of parents for their values and felt a lack of mutual efforts to respect one another, these feelings grew into distress, anger and hostility.

In summary, this article aimed to show the unique situation of the ICT between secular therapists and UO clients, focusing on the less frequently presented therapists’ side of the therapeutic encounter, caused by extreme circumstances in the socio-political arena. These circumstances created an unusual balance of power where the minority holds crucial power over the majority. The secular majority is not seen as *supreme* but is decried, while on the other hand, the UO community is totally dependent on the secular sector that fully finances their lives. Secular therapists, aiming for cultural competence with UO clients, examine their biases and face conflicting values. In this process they encounter challenges when a client from the Haredi sector displays hostility, leading to potential dangers in the cultural intersection, but also potential opportunities for mutual collaboration. While this paper emphasized more the complexity and its less discussed aspects and challenges, further research is required to capture the experience of this unique encounter between the secular therapist and the Haredi clients, demonstrating the nuances of the extreme positions these two cultures hold. As most studies focus on the Haredi clients’ attitudes and needs (Bilu and Witztum, 1993; Bilu and Witztum, 1995; Margolese, 1998; Keidar et al., 2022), future research should also explore secular therapists’ experiences with UO clients. Since an insight into secular therapists’ perspectives is essential for developing effective and inclusive ICT, research should focus on the way the secular therapists’ personal values and their concept of the UO community influence the therapeutic encounter with Haredi clients. Research should also explore the way challenges faced by secular therapists working with UO clients influence their self-perception of professionalism, competence, skills and confidence.

## Author contributions

ED: Conceptualization, Writing – Original Draft Preparation, Writing– Review & Editing. ST: Writing – review & editing. JD: Writing – review & editing, Project Administration, Supervision.

## References

[ref1] Agoro O. (2019). “Who’s being assessed? Post-modernism and intercultural therapy assessments: a synergetic process” in Intercultural therapy: challenges, insights, and developments. eds. Ababio B. Littlewood R. (London and New York: Routledge), 24–39.

[ref2] American Psychological Association. (2010). Ethical principles of psychologists and code of conduct (2002, amended June 1, 2010). Available at: http://www.apa.org.ezproxy.haifa.ac.il/ethics/code/index.aspx.

[ref3] Bagno-Moldavsky O. (2016). “The IDF and the ultra-orthodox: economic aspects of conscription” in Military Service in Israel: challenges and ramifications. eds. Elran M. Sheffer G. (Tel Aviv: Institute for National Security Studies), 93–101.

[ref4] Band-Winterstein T. Freund A. (2015). Is it enough to ‘speak Haredi’? Cultural sensitivity in social workers encountering Jewish ultra-orthodox clients in Israel. Br. J. Soc. Work. 45, 968–987. doi: 10.1093/bjsw/bct167

[ref5] Bilu Y. Witztum E. (1993). Working with Jewish ultra-orthodox patients: guidelines for a culturally sensitive therapy. Cult. Med. Psychiatry 17, 197–233. doi: 10.1007/BF01379326, PMID: 8222706

[ref6] Bilu Y. Witztum E. (1995). Between sacred and medical realities: culturally sensitive therapy with Jewish ultra-orthodox patients. Sci. Context. 8, 159–173. doi: 10.1017/s0269889700001939, PMID: 11639652

[ref7] Brown B. (2017). The Haredim, a guide to their beliefs and sectors. Tel Aviv: Am oved, 275.

[ref8] Caplan K. (2006). “Haredim and western culture: a view from both sides of the ocean” in Middle eastern societies and the west: accommodation or clash of civilizations? ed. Litvak M. (Tel Aviv: The Moshe Dayan Center), 269–289.

[ref9] Caplan K. (2007). Internal popular discourse in Israeli Haredi society. Jerusalem: The Zalman Shazar Center for Jewish History.

[ref10] Caplan K. (2017). The importance of historical perspectives of Israel’s ultra-orthodox society. Dem Cult 17, 267–291.

[ref11] Caplan K. Stadler N. (2012). From survival to consolidation: Changes in Israeli Haredi society. Jerusalem, Israel: Van Leer Institute Press and Hakibbutz Hameuchad, 176–193.

[ref12] Chang M. (2021). Intercultural therapy: challenges, insights, and developments. Body Mov. Dance Psychother. 16, 166–169. doi: 10.1080/17432979.2021.1883739

[ref13] Cincotta R. Kaufmann E. (2009). The changing face of Israel. Foreign policy. Available at: https://foreignpolicy.com/2009/06/01/the-changing-face-of-israel/ (Accessed December 10, 2023).

[ref14] Cockersell P. (2019). “Intercultural psychotherapy, intracultural psychotherapy, or just good psychotherapy?” in Intercultural therapy: challenges, insights, and developments. eds. Ababio B. Littlewood R. (London and New York: Routledge), 94–104.

[ref15] Cohen A. (2006). Jews non-Jews: Israeli Jewish identity and the challenge of expanding the Jewish nationality. Jerusalem: Shalom Hartman Ins. Keter Books Ltd.

[ref16] Coleman-Brueckheimer K. Spitzer J. Koffman J. (2009). Involvement of rabbinic and communal authorities in decision-making by Haredi Jews in the UK with breast cancer: an interpretative phenomenological analysis. Soc. Sci. Med. 68, 323–333. doi: 10.1016/j.socscimed.2008.10.003, PMID: 19019515

[ref17] Doron E. (2020). Art therapy with Jewish ultra-orthodox children: unique characteristics, benefits, and conflicts. Front. Psychol. 11:598917. doi: 10.3389/fpsyg.2020.598917, PMID: 33192946 PMC7652821

[ref18] Dwairy M. A. (1998). Cross-cultural counseling: the Arab-Palestinian case. New York, NY: Haworth Press.

[ref19] Elimelech V. (2012). “The “other” in Haredi society as it is shown through Haredi films” in From survival to establishment: changes in Haredi society and research. eds. Caplan K. Stadler N. (Ra'anana: The Van Leer Institute), 116–137.

[ref20] Eshet I. (2010). The relations between psychological treatment and Judaism [Heb]. Hebrew Psychology. Available at: https://www.hebpsy.net/me_article.asp?article=2474

[ref21] Farook M. W. (2018). The state of multicultural counseling competencies research. Psychot Bull 53, 48–58.

[ref22] Finkelman Y. (2014). The ambivalent Haredi Jew. Israel Stud. 19, 264–293. doi: 10.2979/ISRAELSTUDIES.19.2.264

[ref23] Flaskas C. (2012). “What would (or can) I know? Reflections on the conditions of knowing and understanding in inter-cultural therapy” in Culture and reflexivity in systemic psychotherapy: mutual perspectives. ed. Krause B. (London: Karnac), 53–70.

[ref24] Frankel J. (2002). Exploring Ferenczi's concept of identification with the aggressor: its role in trauma, everyday life, and the therapeutic relationship. Psychoanal. Dialogues 12, 101–139. doi: 10.1080/10481881209348657

[ref25] Freud Z. (1927) in The future of an illusion. ed. Strachey J. (New York: W. W. Norton & Company), 1961.

[ref26] Freund A. Band-Winterstein T. (2013). Between tradition and modernity: social work-related change processes in the Jewish ultra-orthodox society in Israel. Int. J. Intercult. Relat. 37, 422–433. doi: 10.1016/j.ijintrel.2012.10.003

[ref27] Freund A. Band-Winterstein T. (2017). Cultural psychiatry: a spotlight on the experience of clinical social workers’ encounter with Jewish ultra-orthodox mental health clients. Community Ment. Health J. 53, 613–625. doi: 10.1007/s10597-016-0056-9, PMID: 27722905

[ref28] Frosh S. (2004). “Religious influences on parenting” in Handbook of parenting. eds. Hoghughi M. S. Long N. (London, Thousand oaks, New Delhi: Sage Publications), 98–109.

[ref29] Gelso C. J. (2011). The real relationship in psychotherapy: the hidden foundation of change. Washington, DC: American Psychological Association.

[ref30] Goodman Y. Witztum E. (2002). Cross-cultural encounters between care providers: rabbis’ referral letters to a psychiatric Clinic in Israel. Soc. Sci. Med. 55, 1309–1323. doi: 10.1016/S0277-9536(01)00278-7, PMID: 12231011

[ref31] Gopalkrishnan N. (2018). Cultural diversity and mental health: considerations for policy and practice. Front. Public Health 6:179. doi: 10.3389/fpubh.2018.00179, PMID: 29971226 PMC6018386

[ref32] Greenberg D. Witztum E. (2013). Challenges and conflicts in the delivery of mental health services to ultra-orthodox Jews. Asian J. Psychiatr. 6, 71–73. doi: 10.1016/j.ajp.2012.10.008, PMID: 23380322

[ref33] Guzder J. Rousseau C. (2013). A diversity of voices: the McGill ‘working with culture’ seminars. Cult Med Psych 37, 347–364. doi: 10.1007/s11013-013-9316-0, PMID: 23549711

[ref34] Haimovich M. Leiser D. (2017). Ultra-orthodox Jewish perceptions of psychotherapy and psychopathology, BBD, 32. Available at: https://www.researchgate.net/publication/312937018

[ref35] Halstead M. (1995). Voluntary apartheid? Problems of schooling for religious and other minorities in democratic societies. Philos. Educ. 29, 257–272. doi: 10.1111/j.1467-9752.1995.tb00358.x

[ref36] Hess E. (2018). Authority, psychotherapy and the Authority of the Therapist in the religious Haredi community. Am. J. Psychoanal. 78, 137–158. doi: 10.1057/s11231-018-9137-6, PMID: 29662103

[ref37] Hess E. Pitariu H. (2011). Psychotherapy of ultra-orthodox Jews in Israel – a qualitative assessment of conflicts and reconciliations. Eur. J. Psychol. 7, 502–533. doi: 10.5964/ejop.v7i3.146

[ref38] Hoffman S. Ben Shalom H. (2014). “Reflections on working at an ultra-orthodox mental health clinic” in Reader for the orthodox Jewish psychotherapist: case studies and contemporary response. ed. Hoffman S. (NY: Golden Sky), 43–51.

[ref39] Hoffman S. Rossman L. L. (2012). Psychotherapy and Judaism. New York: Golden Sky Books.

[ref40] Jeraisy A. (2013). “Psychosocial therapy in Arab society” in Social work in Israel. eds. Hovav M. Levental M. Katan Y. (Bnei Brak, Israel: United Kibbutz Publisher), 506–526.

[ref41] Kareem J. (1992). “The Nafsiyat intercultural therapy Centre: ideas and experience in intercultural therapy” in Intercultural therapy: themes, interpretations and practice. eds. Kareem J. Littlewood R. (Oxford: Blackwell Scientific Publications), 14–37.

[ref42] Keidar L. Regev D. Snir S. (2021). Non-Haredi arts therapists’ perceptions of therapy with ultra-orthodox children. Front. Psychol. 12:599872. doi: 10.3389/fpsyg.2021.599872, PMID: 33613383 PMC7889799

[ref43] Keidar L. Snir S. Regev D. Keidar E. (2022). Ultra-orthodox parents’ perceptions of arts therapies for their children. Children 9:1576. doi: 10.3390/children9101576, PMID: 36291512 PMC9599959

[ref44] Kirmayer L. J. (2011). Multicultural medicine and the politics of recognition. J. Med. Philos. 36, 410–423. doi: 10.1093/jmp/jhr024, PMID: 21804073

[ref45] Koç V. Kafa G. (2019). Cross-cultural research on psychotherapy: the need for a change. J. Cross-Cult. Psychol. 50, 100–115. doi: 10.1177/0022022118806577

[ref46] Littlewood R. Ababio B. (2019). “Introduction: process and development in intercultural psychotherapy” in Intercultural therapy: challenges, insights, and developments. eds. Ababio B. Littlewood R. (London and New York: Routledge), 1–15.

[ref47] Malach G. Cahaner L., (2018). Annual Haredi Society in Israel: 2018. The Israel democracy institute and Jerusalem institute for policy research (16). Available at: https://www-idi-org-il.ezproxy.haifa.ac.il/haredi/2018/?chapter=25149

[ref48] Malach G. Cahaner L. (2022). Annual statistical report on ultra-orthodox (Haredi) society in Israel. The Israel democracy institute. Available at: https://en-idi-org-il.ezproxy.haifa.ac.il/media/20567/annual-statistical-report-on-ultra-orthodox-haredi-society-in-israel-2022-executive-summary.pdf

[ref49] Margolese H. C. (1998). Engaging in psychotherapy with the orthodox Jew: a critical review. Am. J. Psychother. 52, 37–53. doi: 10.1176/appi.psychotherapy.1998.52.1.37, PMID: 9553639

[ref50] Montgomery A. Ventriglio A. Bhugra D. (2020). “Standards in intercultural psychotherapy” in Intercultural psychotherapy. eds. Schouler-Ocak M. Kastrup M. (Cham: Springer).

[ref51] Nashef Y. (2008). “The social – political context in therapist-patient relations, guided guide, in Arab Jewish dyad” in Educational psychology in a multicultural society. ed. Vail G. (Jerusalem, Israel: Department of Publications Ministry of Education), 127–145.

[ref52] Pew Research Center. (2016). Israel’s religiously divided society. Available at: https://www.pewresearch.org/religion/2016/03/08/israels-religiously-divided-society/ (accessed December 10, 2023)

[ref53] Rober P. De Haene L. (2014). Intercultural therapy and the limitations of a cultural competency framework: about cultural differences, universalities and the unresolvable tensions between them. J. Fam. Ther. 36, 3–20. doi: 10.1111/1467-6427.12009

[ref54] Rube D. M. Kibel R. N. (2004). The Jewish child, adolescent, and family. Child Adolesc. Psychiatr. Clin. N. Am. 13, 137–147. doi: 10.1016/S1056-4993(03)00076-2, PMID: 14723305

[ref8001] Sagi A. (2006). The Jewish Israeli journey. Jerusalem (In Heb.): Shalom Hartman Institute.

[ref55] Schnall E. Kalkstein S. Gottesman A. Feinberg K. Schaeffer C. B. Feinberg S. S. (2014). Barriers to mental health care: a 25-year follow-up study of the orthodox Jewish community. J. Multicult. Couns. Dev. 42, 161–173. doi: 10.1002/j.2161-1912.2014.00052.x

[ref56] Sue S. Zane N. Hall G. C. N. Berger L. K. (2009). The case for cultural competency in psychotherapeutic interventions. Annu. Rev. Psychol. 60, 525–548. doi: 10.1146/annurev.psych.60.110707.163651, PMID: 18729724 PMC2793275

[ref57] Tang N. M. Gardner J. (2014). “Interpretation of race in the transference: perspectives of similarity and difference in the patient/therapist dyad” in Race, culture and psychotherapy. eds. Moodley R. Palmer S. (London: Routledge), 89–99.

[ref58] Zicherman H. (2014). Black blue white: a journey into the Haredi Society in Israel. Tel Aviv: Chemed Corp.

[ref59] Zicherman H. Cahaner L. (2012). Modern ultra-orthodoxy: the emergence of a Haredi middle class in Israel: The Israel Democracy Institute.

[ref60] Zoabi H. (2015). Culture sensitive intervention: the intercultural model – the case of the Arab society Efshar. J. Educ. Soc. Work. 25, 5–7.

